# Bilirubin-microbiota interaction: molecular mechanisms and therapeutic strategies in neonatal jaundice

**DOI:** 10.3389/fmicb.2025.1749152

**Published:** 2026-01-13

**Authors:** Wenlong Yan, Ning Du, Kun Zhang, Pingping Yang, Jing Guo, Lingfen Xu

**Affiliations:** 1 Department of Pediatrics, Shengjing Hospital of China Medical University, Shenyang, Liaoning, China; 2 The Center for Pediatric Liver Diseases, Children’s Hospital of Fudan University, Shanghai, China

**Keywords:** bilirubin, bilirubin encephalopathy, gut microbiota, hyperbilirubinaemia, short-chain fatty acids (SCFAs)

## Abstract

Recent studies have revealed a complex interplay between bilirubin metabolism and the gut microbiota. Bilirubin functions as a potent antioxidant and signaling molecule in humans, and its concentration-dependent effects on distinct microbial taxa indicate that the compound exerts selective pressure on the gut ecosystem. The gut microbiota modulates bilirubin metabolism by altering intestinal pH, producing and activating Bilirubin metabolic enzyme, and bile acids. Because perturbations in bilirubin handling are especially common—and potentially neurotoxic—in neonates, a concise synthesis of recent progress is warranted. Here we review how microbial communities reshape bilirubin flux, how bilirubin and its conjugates, in turn, sculpt microbial ecology, and how the dynamic equilibrium between conjugated and unconjugated bilirubin in hyperbilirubinaemia is influenced by the microbiome. Regulating gut microbiota to accelerate bilirubin clearance or attenuate its toxicity may therefore offer a tractable therapeutic avenue.

## 1 Introduction

Neonatal hyperbilirubinaemia reflects two distinct clinical realities. One is physiological jaundice, a common condition driven by transient accumulation of UCB due to immature liver function (Coraizin et al., 2024). The other is cholestatic disease, marked by elevated levels of conjugated bilirubin (CB)—a red flag for underlying hepatobiliary pathology. Phototherapy remains the primary intervention for reducing UCB. Yet its benefits decline after 48 h of treatment, with diminishing returns despite continued exposure (Kuitunen et al., 2024). Prolonged use also brings risks. These include skin sensitivity to light, potential retinal damage if eye protection fails, and disruption of the early gut microbiome (Faulhaber et al., 2019). In vulnerable infants, such dysbiosis may weaken intestinal defenses, possibly increasing the risk of necrotizing enterocolitis (Li et al., 2023). The challenge grows when breastfeeding-associated jaundice overlaps with signs of cholestasis. What appears to be a benign course can hide serious liver dysfunction, leading to delayed diagnosis and missed opportunities for early care (Feldman and Sokol, 2020). Recent insights have redefined the gut microbiota not just as a bystander but as an active metabolic partner—a kind of symbiotic organ that helps regulate host physiology (Leviatan and Segal, 2020). Within the enterohepatic circulation, certain gut bacteria produce β-glucuronidase, an enzyme that deconjugates excreted bilirubin, allowing it to be reabsorbed and prolonging its presence in the bloodstream (Hamoud et al., 2018). At the same time, microbial 7α-dehydroxylase alters bile acid composition, which may indirectly influence bilirubin metabolism and liver signaling. In our preclinical studies using neonatal models, administering probiotics reduced serum UCB levels. This effect appeared linked to direct binding of bilirubin by bacterial cells in the gut lumen (Jiayi et al., 2024). We also observed better preservation of the intestinal barrier, suggesting these microbes may protect against phototherapy-induced gut injury.

In this review, we focus on elucidating the key molecular mediators of the microbiota-bilirubin interplay and evaluate the translational feasibility of precision microbial therapies for the management of hyperbilirubinemia.

## 2 The enterohepatic circulation of bilirubin

Our red blood cells break down as they age. Inside the spleen, bone marrow, and liver, an enzyme called haem oxygenase opens up the “heme” ring from hemoglobin. This process creates biliverdin, which is immediately turned into UCB by another enzyme, biliverdin reductase (Hirayama et al., 2022; Amirneni et al., 2020). This UCB travels in the bloodstream, attached to a protein called albumin. The liver cells take it up. Inside these cells, an enzyme named UGT1A1 attaches a glucuronic acid molecule to UCB. This changes it into CB, which can dissolve in water. The liver then pushes this CB into bile using special transport pumps, mainly MRP2 and BSEP, so it can leave the body (Hirayama et al., 2022; Amirneni et al., 2020). When the CB reaches the small intestine, gut bacteria get involved. Their enzymes, called β-glucuronidases, chop off the glucuronic acid, turning CB back into UCB. Another bacterial enzyme, bilirubin reductase, then breaks this UCB down further into a group of colorless compounds called urobilinogens. Finally, these are oxidized into the brown pigments (stercobilin) that give stool its color. Only a tiny amount of these breakdown products gets reabsorbed back into the body (Hamoud et al., 2018; Hall et al., 2024). The community of gut microbes actively fine-tunes this system. For example, when certain bacteria like *E. coli* grow, they produce more succinate. This succinate can signal the liver to make more of the UGT1A1 enzyme. On the other hand, common bacterial products called short-chain fatty acids make the intestinal contents more acidic. This acidity can weaken the bond between UCB and albumin, which changes how bilirubin moves between the blood and the bile (Li et al., 2020; Duan et al., 2021).

## 3 Microbial regulation of the bilirubin axis

The aryl-hydrocarbon receptor (AHR) integrates host–microbial crosstalk (Zhang et al., 2024); microbially derived tryptophan metabolites serve as its principal endogenous agonists (Wrzosek et al., 2021; Stockinger et al., 2021). Dysbiosis that depletes these ligands lowers hepatic AHR activation and attenuates albumin-mediated UCB transport, precipitating unconjugated hyperbilirubinaemia (Chen et al., 2022).

β-Glucuronidase (GUS), the gatekeeper enzyme that re-activates bilirubin in the gut, is overwhelmingly of bacterial origin. Metagenomic surveys of murine intestines attribute 60% of GUS genes to Firmicutes and 21% to Bacteroidetes (Creekmore et al., 2019). Early culture-based studies identified *Clostridium ramosum*, *C. perfringens*, *C. difficile*, and *Bacteroides fragilis* as efficient CB-hydrolysers; the subsequent reduction of bilirubin’s conjugated double bonds is catalysed by the strictly anaerobic bilirubin reductase (BilR) (Hall et al., 2024; Harvent and Devuyst, 2024).

Although these pioneer isolates hinted at microbial bilirubin handling, the molecular basis of pigment reduction remained unresolved until the recent biochemical definition of BilR. The enzyme displays narrow substrate specificity for bilirubinoid pigments and a restricted phylogenetic footprint confined to selected anaerobic commensals. This step is rate-limiting for urobilinogen formation and, by extension, for faecal pigment output.

**Figure 1:**
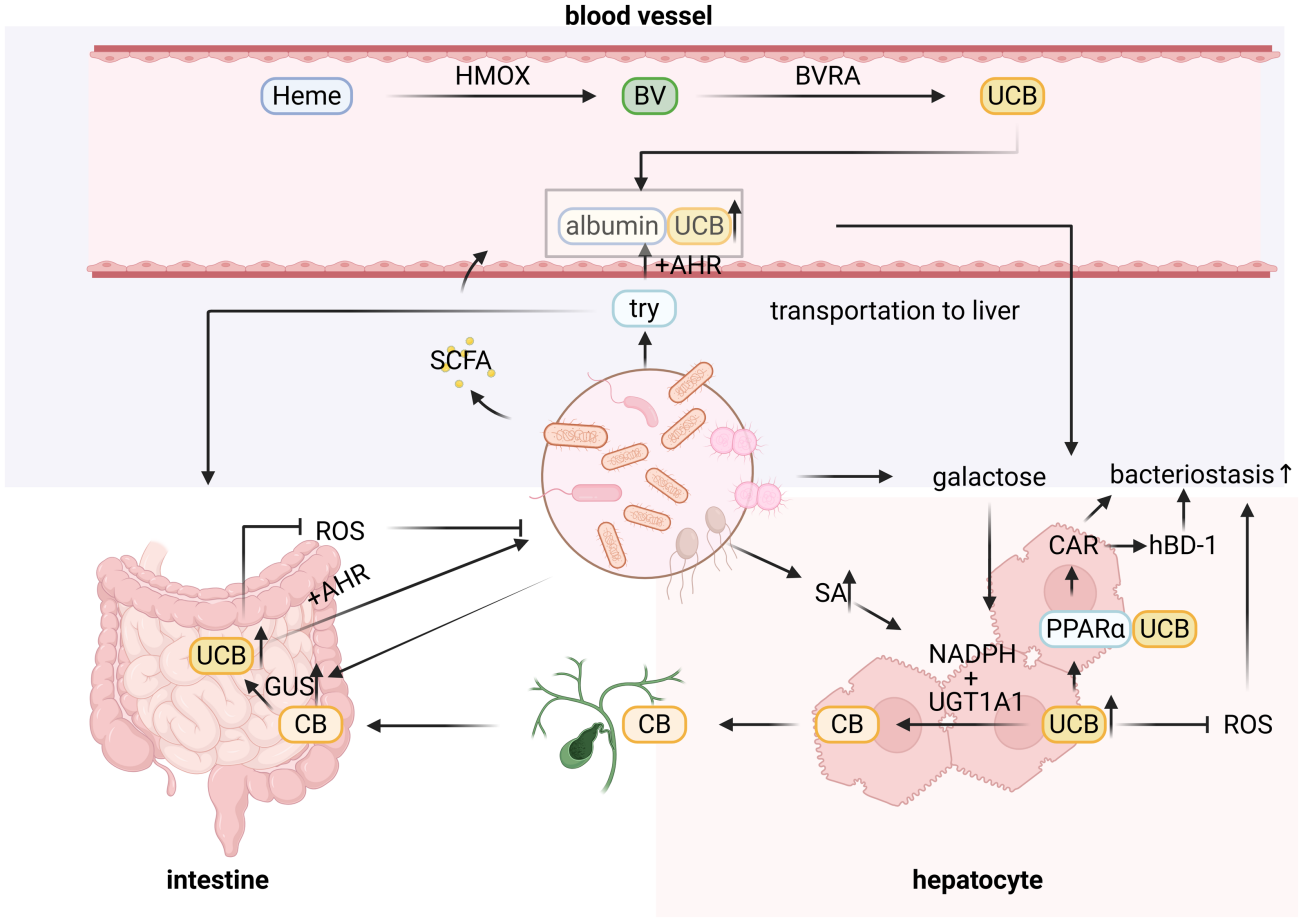
The interaction between intestinal microbiota and bilirubin, and the possible pathways for UCB elevation due to intestinal microbiota disturbance, include the effect of the metabolite tryptophan (Try) on UCB transport efficiency, the reduction of UCB transformation in hepatocytes by succinic acid (SA), and the increase of GUS enzyme production to increase CB transformation. Heme, hematin; HMOX, heme oxygenase; BV, biliverdin; BVRA, biliverdin reductase A; SCFA, short-chain fatty acids; ROS, reactive oxygen species; AHR, aryl hydrocarbon receptor; CAR, constitutive androstane receptor; hBD-1, hydroxyl betaine-1 receptor; PPAR, peroxisome proliferator-activated receptor.

Consistent with this paradigm, neonates harbouring an immature, Clostridia-rich but BilR-poor microbiota exhibit sustained UCB accumulation. BilR prevalence is highest among adult Firmicutes, yet the gene is markedly under-represented in newborns and in patients with inflammatory-bowel disease. Thus, microbial BilR activity constitutes a previously unappreciated checkpoint in bilirubin homeostasis, linking early-life dysbiosis to jaundice risk. Whether BilR deficiency directly heightens neurotoxicity remains untested, and therapeutic augmentation of the enzyme has yet to enter clinical exploration (Figure 1).

## 4 The influence of bilirubin on the intestinal microbiota

In recent years, an increasing number of studies have shown that bilirubin is not only the end product of the catabolism of hemoglobin, but also a bioactive molecule with strong antioxidant activity (Weaver et al., 2018; Huang et al., 2022). Its unique biochemical characteristics enable UCB to exert differentiated regulatory effects on different microbial communities based on differences in concentration and local microenvironment. *In vitro* bacterial culture studies have confirmed that UCB can interfere with the respiratory and carbohydrate metabolism processes of bacteria (Nobles et al., 2013). It is particularly worth noting that UCB shows potential inhibitory effects on some Gram-positive bacteria, such as *Enterococcus faecalis*, *Bacillus cereus*, *Staphylococcus aureus*, and *Streptococcus agalactiae*, etc. On the contrary, UCB seems to have a protective effect on Gram-negative bacteria, and its antioxidant properties can help pathogens such as *enterohemorrhagic Escherichia coli* resist reactive oxygen species (ROS) -mediated oxidative stress. Interestingly, some studies have also pointed out that physiological hyperbilirubinemia may inhibit the growth of pathogenic bacteria such as Group B Streptococcus and potentially exert a protective effect against shock induced by endotoxin of Gram-negative bacteria (Hansen et al., 2018). However, in the state of severe unconjugated hyperbilirubinemia, endotoxin was instead proven to enhance cytotoxicity, suggesting a more complex interaction between bilirubin levels and host-microbial immune dynamics (Ngai and Yeung, 1999). More importantly, UCB has been confirmed as a direct agonist of peroxisome proliferator-activated receptor α (PPARα) (Marghani et al., 2022; Gordon et al., 2020). The activation of PPARα is associated with a reduction in neutrophil infiltration and can inhibit the increase in intestinal permeability caused by inflammatory stimulation, thereby helping to maintain intestinal microbiota homeostasis (Manickam et al., 2020). In addition, UCB is a potent endogenous activator of aromatic hydrocarbon receptors (AHR), which play a crucial role in maintaining the integrity of the mucosal barrier and immune tolerance in the intestinal microenvironment (Stockinger et al., 2021). In the state of cholestasis, UCB may indirectly activate constitutive androgen receptors (CARS), thereby upregulating the expression of human β-defensin-1 (hBD-1). This antimicrobial peptide can enhance the liver’s defense ability against pathogens such as *Escherichia coli*, highlighting the possible protective effect of bilirubin signaling in liver immunity (Klag et al., 2018).

## 5 The interaction between bilirubin and the intestinal microbiota in disease

Elevated levels of UCB can result from increased production, impaired transport or binding, and reduced hepatic clearance. Among these, physiological jaundice is the most common clinical manifestation in neonates.

In early life, the intestinal microbiota is enriched with *Clostridium ramosum*, *C. perfringens*, and *C. difficile*, while the abundance of *Bacteroides fragilis* remains low. This microbial imbalance limits the conversion of CB into urobilinogen, a process mainly mediated by BilR. As a result, urinary bilirubin becomes detectable only around the fifth day of life (Vítek et al., 2000). The delayed metabolism leads to the accumulation of CB in the intestine, where β-glucuronidases (GUS), which are highly active under these conditions, hydrolyse CB back to UCB. This UCB is then reabsorbed via enterohepatic circulation, contributing to the development of neonatal jaundice.

Pathological hyperunconjugated bilirubinaemia is primarily caused by hemolytic jaundice, which shares similar onset features with physiological jaundice. In this condition, excessive UCB is released into the bloodstream due to accelerated erythrocyte destruction, surpassing the hepatic capacity for conjugation in neonates. This overload disrupts the normal transport and clearance of UCB, necessitating medical intervention (Ou and Tsai, 2022).

Another important subtype of hyperunconjugated bilirubinaemia is breast milk jaundice (BMJ), which has a complex etiology potentially involving multiple metabolic pathways. Alterations in the composition and function of the gut microbiota following breastfeeding have been implicated as a key pathogenic mechanism.

Studies (Duan et al., 2021; Ou and Tsai, 2022; Jones and Neish, 2021) have demonstrated that infants with BMJ exhibit significantly increased abundances of *Klebsiella* and *Escherichia coli*, along with decreased levels of *Bifidobacterium*, *Enterococcus*, and *Streptococcus*. Notably, *Klebsiella* species possess β-glucuronidase activity, which enhances the hydrolysis of CB and contributes to elevated UCB levels (Guo et al., 2023). This increase may exceed the liver’s reabsorption capacity.

The reduction in *Bifidobacterium* abundance may impair the expression or activity of UGT1A1. Research (Thongaram et al., 2017) indicates that *Bifidobacterium* participates in galactose metabolism, a precursor for UDP-glucuronic acid—the substrate required for UGT1A1 activity. Thus, diminished *Bifidobacterium* levels impair hepatic UCB conjugation. Additionally, lower *Bifidobacterium* abundance decreases enterohepatic cycling and intestinal pH, further promoting GUS activity and enhancing UCB hydrolysis and reabsorption (Deshmukh et al., 2019).

The decline in *Enterococcus* and *Streptococcus* populations reduces the production of short-chain fatty acids (SCFAs), particularly propionate and acetate. These metabolites are known to enhance UCB–albumin binding; therefore, their depletion may impair UCB transport and contribute to its systemic accumulation (Novák et al., 2020).

Intestinal microbes also regulate host nuclear receptors such as peroxisome proliferator-activated receptors (PPARs) (Yu et al., 2020). Activation of PPARs can further modulate xenobiotic receptors, including the constitutive androstane receptor (CAR) and the pregnane X receptor (PXR) (Kobayashi et al., 2022), thereby downregulating the expression of bilirubin-metabolizing enzymes such as Cyp3a11. A decrease in *Enterococcus* abundance may exacerbate this suppression, leading to enhanced CB accumulation and UCB reabsorption (Weber et al., 2024). Furthermore, early antibiotic use—particularly combinations such as ampicillin and gentamicin—has been shown to significantly reduce gut microbiota diversity and deplete beneficial bacterial taxa. For example, in preterm infants treated with this regimen, the relative abundance of Enterobacteriaceae increases, while that of *Bifidobacterium* and *Lactobacillus* decreases. Such dysbiosis may enhance the enterohepatic circulation of bilirubin, resulting in elevated serum bilirubin levels and prolonged jaundice (Su et al., 2024).

Clinically, bilirubin encephalopathy can present as abnormal muscle tone, hearing impairment, ocular motility dysfunction, delayed cognitive development, and in severe cases, it can lead to death (Qian et al., 2022; Par et al., 2023). In recent years, an increasing amount of evidence has shown that the gut microbiota not only participates in the metabolism of bilirubin but may also influence the occurrence and progression of bilirubin encephalopathy through the “gut-brain axis,” which provides a new perspective for the prevention and treatment intervention of the disease.

At physiological concentrations, UCB exerts neuroprotective effects by regulating oxidative stress and maintaining neuronal function (Abe et al., 2021). However, when UCB levels abnormally increase, it can cause irreversible neurological damage through multiple mechanisms such as peroxidation-reduction imbalance, DNA damage, mitochondrial dysfunction, and activation of apoptotic signaling pathways (Abe et al., 2021; Rawat et al., 2018). Specifically, bilirubin can increase mitochondrial membrane permeability, disrupt the electron transport chain, and promote excessive generation of reactive oxygen species (ROS), ultimately leading to mitochondrial swelling, loss of membrane potential, and cellular energy depletion. These changes further trigger an imbalance in caspase-dependent and non-dependent apoptotic pathways, including abnormal regulation of caspase-3, caspase-9, and members of the Bcl-2 family, ultimately leading to irreversible neuronal death (Datta et al., 2023).

**Figure 2:**
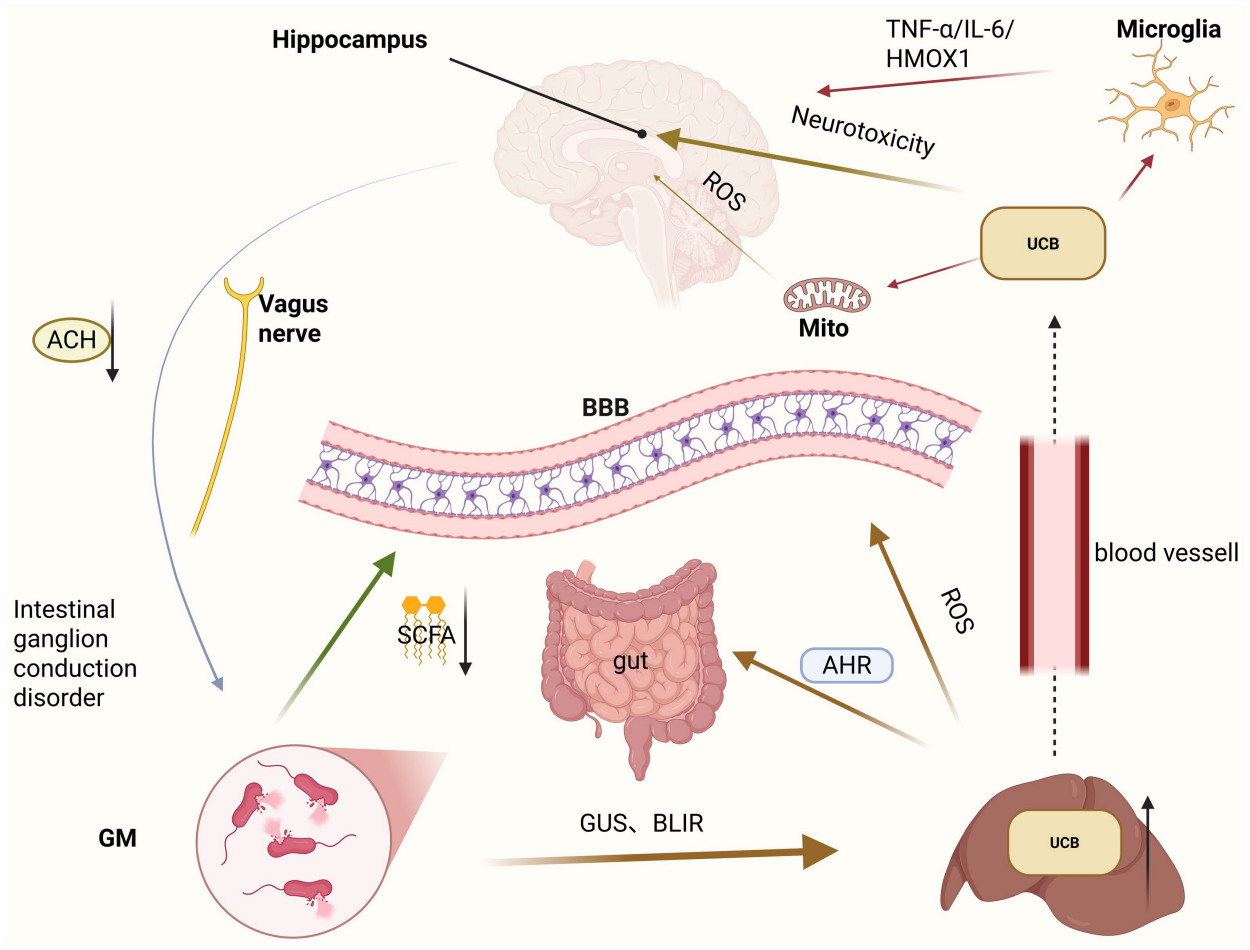
In hyperbilirubinemia, bilirubin, gut microbiota, and the cerebral nervous system constitute a complex liver-gut-brain axis. Excessive UCB can damage the intestinal barrier, which leads to a reduction in SCFAs. The synergistic effect of the two jointly promotes UCB to pass through the blood–brain barrier, thereby activating microglia and causing neuroinflammatory responses. Destroying mitochondria increases cell apoptosis and its own cytotoxic substances, which in turn causes bilirubin encephalopathy. This, in turn, damages the vagus nerve, leading to a reduction in ACH, which in turn causes intestinal peristaltic nerve disorders, further aggravating the damage to the intestinal barrier and the accumulation of bilirubin. UCB, unconjugated bilirubin; Mito, mitochondrion; BBB, blood–brain barrier; SCFA, short-chain fatty acids; GM, gut microbiome; ACH, acetylcholine.

UCB can also induce astrocytes to release pro-inflammatory cytokines such as interleukin-1β (IL-1β) and tumor necrosis factor-α (TNF-α), thereby enhancing the vulnerability of neurons (Abe et al., 2021; Cui et al., 2021; Kumbhar et al., 2024). Meanwhile, UCB can impair the uptake of glutamic acid by astrocytes, leading to an increase in extracellular glutamic acid levels and exacerbating excitotoxicity. Microglia can be activated through the Toll-like receptor 2 (TLR2) pathway under UCB stimulation, further promoting the neuroinflammatory response, increasing the secretion of TNF-α and IL-6, and upregulating the expression of the oxidative stress-related gene HMOX1 (encoding heme oxygenase-1) (Kumbhar et al., 2024). UCB may also damage the integrity of the blood–brain barrier by disrupting tight junction proteins, increasing its permeability and thereby enhancing neurotoxicity. This effect may have a synergistic amplification effect with coexisting inflammatory mediators, further promoting the development of bilirubin encephalopathy. Severe neonatal jaundice is often accompanied by gut microbiota imbalance, which may exacerbate UCB-induced blood–brain barrier dysfunction. The research (Yang et al., 2019) shows that newborns with severe hyperbilirubinemia accompanied by neurological abnormalities have reduced intestinal microbiota diversity, while bilirubin levels in serum and cerebrospinal fluid increase. Compared with infants with simple hyperbilirubinemia, children with bilirubin encephalopathy have significantly weakened metabolic capabilities of branched-chain amino acids such as alanine, ornithine, isoleucine and leucine (Xie et al., 2023; McCarthy et al., 2019). This metabolic disorder may further increase the permeability of the blood–brain barrier, promoting more unconjugated bilirubin to enter the central nervous system and accelerating the occurrence of kernicterus. In addition, systemic inflammation caused by gut microbiota imbalance may exacerbate nerve damage. For instance, in neonatal hypoxic–ischemic encephalopathy, intestinal dysfunction is associated with the upregulation of Toll-like receptor 4 (TLR4) expression, which may affect the consciousness state of the children (Chen et al., 2025) (Figure 2).

## 6 The application and prospect of microecological therapy in neonatal jaundice

The progress in research on the gut microbiome has drawn much attention to probiotics as an auxiliary management strategy for neonatal jaundice. More and more evidence indicates that the combination of probiotics and phototherapy can produce a synergistic effect, not only significantly shortening the time required for phototherapy but also helping to reduce the hospital stay of children patients (Chen et al., 2017). This provides a new idea for optimizing the existing treatment plans for neonatal jaundice. For instance, both *Lactobacillus salivalis* AP-32 and *Bifidobacterium lactis* CP-9 have been confirmed to promote bilirubin clearance. A randomized controlled trial showed that the average phototherapy time required by infants receiving AP-32 was approximately 7 h less than that of the placebo group, and the CP-9 group also showed a trend of shortened treatment time (Tsai et al., 2025). These two probiotics also significantly enhanced the diversity of the gut microbiota in infants. The abundance of Lactobacillus salivary in the AP-32 group increased significantly, and the level of bifidobacterium was even more prominent in the CP-9 group (Tsai et al., 2025).

However, this field still faces several challenges. First, the issue of safety cannot be ignored. Although the overall incidence of sepsis associated with probiotics is relatively low, the majority of reported cases are concentrated in very low birth weight or premature infants (Feldman et al., 2025), suggesting that the risk has not been completely ruled out in these vulnerable populations. Secondly, most existing studies are confined to a single institution, which may lead to regional or population bias and affect the universality of the conclusions. Third, there is still a lack of consensus on key parameters such as the specificity of probiotic strains, the timing of administration, and the standardization of treatment courses. To clarify these issues, more studies that are well designed and conducted across multiple centers are urgently needed.

In addition to its role in neonatal jaundice, recent studies have gradually revealed that slightly elevated UCB has protective effects on various metabolic and inflammatory diseases. The bidirectional regulatory network formed between bilirubin and the gut microbiota is a complex one that far exceeds the scope of neonatology. For instance, the mechanism by which UCB inhibits the activity of intestinal proteases is an important entry point for understanding intestinal health and diseases. Early studies have suggested that UCB in the intestinal lumen can inhibit protease activity and may play a protective role in maintaining intestinal homeostasis (Ozaka et al., 2021). The significance of this mechanism lies in the fact that excessive protease activity can damage the intestinal mucosal barrier and participate in pathological processes such as inflammatory bowel disease (IBD). The absence of the protective effect that is mediated by UCB may be related to the occurrence and development of IBD (Qin, 2002). In response to this, the expression of bilirubin reductase genes in the intestines of IBD patients is decreased (Hall et al., 2023), and the abundance of bacteria capable of metabolizing conjugated bilirubin (such as *Ruminococcus* and *Bifidobacterium*) is also generally reduced (Vich Vila et al., 2023).

Regulating the activity of bacterial-derived β-glucuronidase (GUS) provides a potential new target for managing diseases such as irritable bowel syndrome (IBS) (He et al., 2019a; He et al., 2019b). Recently, the study (Edwinson et al., 2022) conducted metabolomics analysis and found that there was a difference in fecal GUS activity between IBS patients and healthy controls. They also confirmed that only free bilirubin produced by microbial GUS action can effectively inhibit protease function. On the other hand, the activation effect of UCB on cell receptors such as PPARs may play a beneficial role in metabolic regulation by promoting fatty acid oxidation, enhancing energy expenditure, improving insulin sensitivity, and regulating fat production and inflammatory responses.

## 7 Conclusion

In conclusion, our review highlights the dynamic communication in the bilirubin gut axis. While multiple organs are involved in bilirubin metabolism, the liver and intestine are uniquely interconnected through enterohepatic circulation, playing a crucial role in maintaining bilirubin homeostasis and balancing its beneficial antioxidant effects with potential neurotoxicity. We have shown that the bilirubin microbiota dialogue involves both microbiota dependent and microbiota independent mediators, emphasizing the complexity of this bidirectional interaction. By understanding these pathways, we can anticipate advancements in microbiome targeted therapeutic approaches for neonatal jaundice and related disorders. Ultimately, further research will help to clarify how microbial modulation through probiotics, prebiotics, or enzyme based strategies can regulate bilirubin signaling and neuroprotection, offering prospects for personalized management of hyperbilirubinemia and improved clinical outcomes.
